# Passage of Sanguineous Corpuscles through the Walls of the Vessels, Etc.

**Published:** 1868-10-15

**Authors:** 


### CHICAGO MEDICAL JOURNAL.

*Vol. XXV.--OCTOBER* 15, 1868.—*No.* 20.

### PHYSIOLOGY.

### *Passage of Sanguineous Corpuscles through the walls of the vessels; mechanism of this phenomenon and consequences which result therefrom in relation to the formation of pus, and to absorption in general.*

TRANSLATED EXPRESSLY FOR THIS JOURNAL, BY WALTER HAY, M.D., ASSOCIATE EDITOR.

THE point of departure of this problem is found in the researches of M. Cohnheim into the subject of the passage of sanguineous corpuscles through the vascular parietes without apparent lesion of these parietes. The white corpuscles would constitute pus, and the red would form under the given conditions sanguineous extravasations.

Let us first consider the facts; we will next examine the mechanism by means of which they are produced, and the consequences which may be deduced therefrom.

Mr. Cohnheim exposes to the air the mesentery of a frog paralyzed with woorara. Inflammation is developed, and at the period of oscillation the white corpuscles, which seem glued to the internal face of the wall of the vessel, depress this wall, lodge in sorts of pockets which are dependent from these, and continuing their migration, spread themselves into the surrounding tissues, where they are found in a free state, similar in all respects to what they were in the interior of the vessels.

The passage of the red globules through the vascular parietes has been observed and described by M. Strickler, (of Vienna,) and his pupil, M. Bussak. When they poison frogs they inject under their skins a solution of a tenth part of chloride of sodium, and produce thereby in these animals a scorbutic condition, which is manifested by hæmorrhages into the lungs, the liver, the kidneys, the intermuscular tissue, etc. The two preceding observers have determined that i these conditions the red globules which form the hæmorrhagic foci have traversed the walls of the capillary vessels. They have seen, in fact some of these globules which had remained imprisoned in the wall, half projecting, and as if entangled in the intra parietal opening through which they had found an issue.

M. Bastian has repeated the experiments of the German physicians, and has made them the subject of a communication to the pathological society of London. After having paralyzed a frog by means of a subcutaneous injection of woorara, he tied the femoral vein, and soon the blood is massed in the capillaries and the veins. The following are the consecutive phenomena which he was enabled to observe:

Whilst examining the interdigital membrane the serum is seen to transude and induce a certain amount of œdema. The blood soon begins to oscillate in the vessels, and after fifteen or twenty minutes it is completely arrested in some of the capillaries. The red corpuscles are ranged one against the other, and appear to form a homogeneous cylindrical mass. The stasis extends next to other capillaries, and terminates by involving the veins. At the expiration of forty minutes projections form in the walls of the capillaries, which increase in number and in volume so as to give these walls a tuberculated appearance. Then these projections separate gradually from the capillaries, and are found in the neighboring tissues masses of red corpuscles.

If the ligature of the femoral vein is removed, the circulation is re-established in the capillaries, and it is then possible to see besides the red globules which constituted the aforesaid masses, other globules pursuing the same transit as those, and more or less entangled in the capillary parietes. It is possible thus to follow the globules in all the phases or degrees of their migration. The experiments of M. Bastian are thus confirmatory of those of MM. Cohnheim, Strickler, and Bussak.

Such are the phenomena observed. By what mechanism are they produced, or in other words, how can the white or red sanguineous corpuscles in conditions of inflammation, the scorbutic state, or under compression, traverse the capillary parietes? Three orders of explanation, three hypotheses, are proposed:

The most simple, that which suggests itself to the mind very naturally, is that the vascular walls are perforated with pores in which the corpuscles may engage themselves. Such likewise is the theory adopted by MM. Cohnheim, Recklinghausen, Letzerich, F. Keber, etc. The microscope also seems to justify this manner of viewing the subject. M. F. Keber, indeed, since 1854, and in a very recent treatise designed to recall his first investigations, has described and figured the pores or stomata of the capillary vessels, and the vacuities in the intestinal epithelium. M. Cohnheim, on his side, agreeing with MM. Asby, Eberth, Auerbach and others, admits that the capillaries are formed by the juxtaposition of flattened epithelial cells. Now at the points of reunion of the angles of the cells there would remain orifices, pores, through which the corpuscles placed transversely might become engaged, in consequence of the pressure which they undergo in the vessels.

The second hypothesis has for its advocates MM. Strickler and Bussak. These authors consider the parietes of the capillary vessels as composed of a protoplasma, soft, homogeneous and contractile. This protoplasma possesses the property of giving origin to appendices, filiform prolongations which increase, are hollowed out and form new capillaries in communication with the parent wall. The transit of sanguineous globules does not, therefore, necessitate the pre-existence of pores in the capillary wall; it occurs in consequence of an active function in this very wall. MM. Strickler and Bussak admit that the protoplasma in question comports itself like the cells of connective tissue, and that it can, after the manner of these latter, undergo pathological transformation.

Finally, according to a third hypothesis, sustained by M. Bastian, it is not the capillary walls which are active, but, in fact, the red corpuscles of the blood. These last, indeed, can traverse vascular membranes, in consequence of certain active amæbord movements which may manifest themselves subsequent to alterations in the blood plasma. The phenomena, therefore, may be produced in the same manner as the passage of leucocytes through the venous parietes, as M. Cohnheim has established, or through permeable membranes, as M. Lorbet has demonstrated.

We shall not dwell further upon these different hypotheses; they demonstrate the neces sity of new investigations. However it may be, the facts which they attempt to explain, exist none the less, and the consequences which are deduced from them recall into discussion theories by which other orders of phenomena have been explained.

We have seen already that M. Cohnheim, after having determined the passage of white globules through the vascular walls, was disposed to consider them as constituting, after their escape, pus globules. M. Lorbet shares in the same opinion. M. Lionel Beale, on the contrary, opposes it. According to the English author, the white corpuscle of the blood and the pus corpuscle are by no means one and the same thing. They can not ever be transformed, the one into the other. He adds, that suppuration does not pursue the same process in warm blooded animals as in frogs. His understanding of the genesis of pus is as follows:

The pus corpuscle originates from the normal blastema

(germinal matter) of any tissue or element whatsoever. If this blastema receives an excess of nourishment, it increases, is divided, sub-divided, and gives origin to products which differ by their properties from the primitive elements. Pus may, therefore, originate from the proliferation of all sorts of blastema, of that which is peculiar to nerves, to muscles, to vascular walls, etc., as if that which pertains to epithelial cells or to connective tissue. But according to M. Beale, although he admits the passage of the white globules through the vascular parietes, these globules do not become pus corpuscles; it does not appear to him rational to give a new appellation to an element which has only changed its position.

We are here presented with three or four theories, at least, to explain the formation of pus. According to M. Virchow, and his school, the pus globule originates only from cellular proliferation of connective tissue.

Mr. Beale asserts, as we have seen, that every tissue may participate in this process of proliferation.

According to M. Robin and the French school, pus globules may be developed by genesis in the midst of an exudation or of a blastema.

Finally, is presented a new theory, or rather an old theory rejuvenated, according to which these same globules are only white blood corpuscles, which may have traversed the vascular parietes.

In the face of all these hypotheses, these doubts, it must be understood, that the most elementary facts in pathology are discussed, and that openly in the Academy, the long mooted question is ever agitated, if pus may or may not be re-absorbed in all its elements. It is permitted to hope, however, that the recent researches into the parietes of vessels and of mucous membranes, and a greater number of experiments upon the passage of the blood globules through these walls and membranes, will throw some light upon the manner in which the mechanism of phenomena, so numerous and so varied, which are associated with absorption, is to be comprehended.

It is evident, for example, that if the porosity of the vascular walls, or of the epithelial investment of membranes should receive a definite demonstration, the general opinion upon the absorption of organic elements, and of mineral substances reduced to powder, would be entirely modified.

